# Antithrombotic therapy in peripheral arterial disease

**DOI:** 10.3389/fcvm.2022.927645

**Published:** 2022-10-13

**Authors:** Christine Espinola-Klein, Gerhard Weißer, Volker Schmitt, Melanie Schwaderlapp, Thomas Munzel

**Affiliations:** ^1^Center for Cardiology, Cardiology III–Angiology, University Medical Center of the Johannes Gutenberg University Mainz, Mainz, Germany; ^2^Center for Cardiology, Cardiology I–General and Interventional Cardiology and Intensive Care, University Medical Center of the Johannes Gutenberg University Mainz, Mainz, Germany

**Keywords:** peripheral arterial disease, antithrombotic therapy, antiplatelet drugs, rivaroxaban, major adverse cardiovascular events, major adverse limb events

## Abstract

**Background:**

Patients with peripheral arterial disease (PAD) are at increased risk for major adverse cardiovascular events (MACE) such as cardiovascular death, myocardial infarction, and stroke as well as major adverse limb events (MALE) such as amputation and acute limb ischemia. Therefore, prevention of thrombotic events is crucial to improve the prognosis of PAD patients. This review article concludes current evidence and guideline recommendations about antithrombotic therapy in PAD patients.

Antithrombotic therapy is highly effective to reduce MACE and MALE events in PAD patients. Recently, the concept of dual pathway inhibition (low-dose rivaroxaban plus acetylic salicylic acid (ASA) has been tested in the COMPASS and VOYAGER-PAD trial. Compared to ASA alone dual pathway inhibition was superior to prevent MACE and MALE. After peripheral revascularization, in particular the risk for acute limb ischemia was reduced. In contrast, the risk for major bleeding is increased. Therefore, current guidelines recommend the combination of low-dose rivaroxaban and ASA in PAD patients with low bleeding risk. In patients with high bleeding risk, a single antiplatelet drug (preferable clopidogrel) is indicated. In patients with atherosclerotic vascular disease and indication for oral anticoagulation, no additional antiplatelet drug is necessary, as this would increase the risk of bleeding without improving the prognosis.

**Conclusion:**

Antithrombotic treatment reduces MACE and MALE and is recommended in all patients with PAD. Individual bleeding risk should always be considered based on the current data situation and an individual benefit-risk assessment must be carried out.

## Introduction

Peripheral arterial disease (PAD) has a high prevalence and affects more than 200 million people worldwide (1). Atherosclerosis is a disease of the entire vascular vessel tree and especially patients with PAD are at high risk for polyvascular atherosclerosis (2, 3). Therefore, patients with PAD have high atherosclerotic burden and are at increased risk for cardiovascular events.

Although PAD is common and associated with a poor prognosis, the disease is underdiagnosed and underestimated. In addition, patients with PAD are often treated less consistently compared to other manifestations of atherosclerosis such as coronary artery disease (CAD) (4, 5). In particular, the recommendations of the guidelines are less often implemented in PAD patients compared to patients with CAD (5).

In addition to the consistent management of cardiovascular risk factors, antithrombotic therapy is highly indicated in patients with PAD. Antithrombotic therapy reduces the risk for major adverse cardiovascular events (MACE) such as cardiovascular death, myocardial infarction, and stroke as well as major adverse limb events (MALE) such as amputation and acute limb ischemia (2, 3). Therefore, antithrombotic drugs have a high degree of recommendation in current guidelines (2, 3).

In critical limb ischemia with rest pain or wounds, interventional or surgical revascularization is urgently indicated to prevent major amputation. In patients with intermittent claudication and short walking distance, revascularization is often necessary to maintain the mobility of the patient. But after peripheral revascularization, the risk of acute limb ischemia is increased and antithrombotic drugs are a central component of post-interventional therapy (6, 7).

In patients with high-risk atherosclerosis the combination of low dose rivaroxaban and acetyl salicylic acid (ASA) is superior compared to ASA alone regarding reduction of MACE and MALE (8, 9). But every combination of antiplatelet and anticoagulant drugs can increase bleeding risk (10). Therefore, a careful assessment of thrombotic vs. bleeding risk is necessary for each patient.

The following review article concludes current knowledge and guideline recommendations for antithrombotic therapy in patients with PAD under different clinical situations.

## Antithrombotic therapy

Blood coagulation is a complex process in which platelet activation and fibrin generation play a central role (10). Drugs like aspirin, clopidogrel, prasugrel and ticagrelor are inhibitors of platelet aggregation. Vitamin K antagonists (VKA), Factor X inhibitors (apixaban, edoxaban, rivaroxaban) and dabigatran inhibit fibrin generation.

Usually, antiplatelet drugs are indicated in PAD patients. The main indications for antithrombotic therapy in patients with symptomatic PAD are stable patients and after endovascular or surgical revascularization. In high-risk patients the combination of anticoagulant and antiplatelet dugs is useful and in some patients with PAD full dosage antithrombotic therapy is necessary because of concomitant diseases. Choice of anticoagulant or antiplatelet drug also depends on the clinical situation.

Table 1 summarizes the main studies discussed in the following text. Guideline recommendations based on current evidence are summarized in Table 2.

**Table 1A:** Studies on antithrombotic therapy in patients with stable PAD.

**Study name**	**Patients, number of patients with PAD**	**Comparison**	**Primary endpoint**	**Safety endpoint**
Clopidogrel vs. Aspirin in Patients at Risk of Ischaemic Events (CAPRIE) (11)	Chronic CVD *N* = 6,452 (subgroup PAD)	ASA 325 mg o.d. vs. Clopidogrel 75 mg o.d.	Reduction of MACE Clopidogrel vs. ASA RR 23.8% **(*****P*** **=** **0.01)**	Clopidogrel vs. ASA IHC 0.33 vs. 0.47% (*P* = ns) GIB 0.52 vs. 0.72% (*P* = ns)
Examing Use of Ticagrelor in Peripheral Artery Disease (EUCLID) (12)	Symptomatic PAD *N* = 13,887	Ticagrelor 90 mg b.d. vs. Clopidogrel 75 mg o.d.	Reduction of MACE Ticagrelor vs. Clopidogrel HR [95%CI] = 1.02 [0.92–1.13] (*P* = ns)	Major bleeding Ticagrelor vs. Clopidogrel HR [95%CI] = 1.10 [0.84–1.43] (*P* = ns)
Clopidogrel for High Atherothrombotic Risk and Ischemic Stabilization, Management and Avoidance (CHARISMA) (13)	Chronic CVD *N* = 2,838 (subgroup PAD)	Clopidogrel 75 mg o.d. vs. Placebo in addition to ASA 75-162 mg o.d.	Reduction of MACE Clopidogrel + ASA vs. ASA HR [95%CI] = 0.85 [0.66–1.08] (*P* = ns)	Severe bleeding Clopidogrel + ASA vs. ASA HR [95%CI] = 1.18 [0.86–1.60] (*P* = ns)
Thrombin Receptor Antagonist in Secondary Prevention of Atherothrombotic Ischemic Events (TRA 2P–TIMI 50 trial) (14)	Chronic CVD *N* = 3,787 (subgroup PAD)	Vorapaxar 2.5 mg o.d. vs. Placebo in addition to single or dual antiplatelet therapy	Reduction of MACE Vorapaxar vs. placebo HR [95% CI] = 0.94 [0.78–1.14] (*P* = ns)	Major bleeding Vorapaxar vs. placebo HR [95% CI] = 1.62 [1.21–2.18] **(*****P*** **=** **0.001)**
Prevention of Cardiovascular Events in Patients with Prior Heart Attack Using Ticagrelor Compared to Placebo on a Background of Aspirin (PEGASUS) (15)	1 to 3 years after MI *N* = 1,143 (subgroup PAD)	Ticagrelor 60 mg o.d. or Ticagrelor 90 mg o.d. or placebo in addition to ASA 100 mg o.d.	Reduction of MACE Ticagrelor vs. placebo HR [95% CI] = 1.60 [1.20–2.13] **(*****P*** **=** **0.0013)**	Major bleeding Ticagrelor vs. placebo HR [95% CI] = 1.57 [0.47–5.22] (*P* = ns)
Warfarin Antiplatelet Vascular Evaluation Trial (WAVE) (16)	PAD or carotid stenosis *N* = 1.767 (subgroup PAD, carotid stenosis excluded)	VKA or placebo in addition to single antiplatelet therapy	Reduction of MACE VKA vs. placebo RR [95% CI] = 0.92 [0.73–1.16] (*P* = ns)	Life-threatening bleeding VKA vs. placebo RR [95% CI] = 3.41 [1.84–6.34] **(*****P*** **=** ** <0.001)**
Cardiovascular OutcoMes for People Using Anticoagulation StrategyS (COMPASS) (8, 17)	Chronic CVD *N* = 5,551 (subgroup PAD, carotid stenosis excluded)	ASA 100 mg o.d. or Rivaroxaban 5 g b.d. or ASA 100 mg and Rivaroxaban 2.5 mg b.d.	Reduction of MACE ASA vs. ASA + Rivaroxaban HR [95% CI] = 0.70 [0.56–0.88] **(*****P*** **=** **0.002)**	Major bleeding ASA vs. ASA + Rivaroxaban HR [95% CI] = 2.12 [1.21–3.71] **(*****P*** **=** **0.007)**

Significant P-values are displayed in bold.

**Table 1B:** Studies on antithrombotic therapy in patients after peripheral revascularization.

**Study name**	**Patients, number of patients with PAD**	**Comparison**	**Primary endpoint**	**Safety endpoint**
Management of peripheral arterial interventions with mono or dual antiplatelet therapy (MIRROR) (18)	PAD after peripheral intervention N=80	Clopidogrel 75 mg or placebo in addition to ASA 100 mg	Platelet activation Clopidogrel of placebo ß-TG 365.5 vs. 224.5 **(*****P*** **=** **0.03)** CD40L was 127 and 206.5 **(*****P*** **=** **0.05)**	Bleeding Clopidogrel vs. placebo *N* = 1 vs. *N* = 2 minor bleedings (*P* = ns)
The Sufficient Treatment of Peripheral Intervention by Cilostazol (STOP-IC) (19)	PAD after peripheral intervention *N* = 200	Cilostazol with ASA 100 mg vs. placebo with ASA 100 mg	Restenosis Cilostazol vs. placebo OR [95% CI] = 0.26 [0.13–0.53] **(*****P*** **=** **0.0001)**	Bleeding risk similar in both groups, no detailed information in the publication
Edoxaban in Peripheral Arterial Disease (ePAD) (20)	PAD after peripheral intervention N=203	Edoxaban vs. placebo	Restenosis / reocclusion Edoxaban vs. placebo RR [95% CI] = 0.89 [0.59–1.34] (*P* = ns)	Major bleeding (TIMI) Edoxaban vs. placebo RR [95% CI] = 0.56 [0.19–1.62] (*P* = ns)
Dutch BOA (the Dutch Bypass Oral anticoagulants or Aspirin) (21)	PAD after bypass surgery *N* = 2,690	VKA vs. ASA 80 mg o.d.	Graft occlusion VKA vs. ASA 80 mg HR [95% CI] = 0.95 [0.82–1.11] (*P* = ns)	Major bleeding HR [95% CI] = 1.96 [1.42–2.71] **(*****P*** **<** **0.001)**
Sarac et al. (22)	PAD after bypass surgery *N* = 56	ASA vs. VKA and ASA 325 mg o.d.	Primary graft patency (3 years) VKA vs. VKA and ASA 51 vs. 74% **(*****P*** **=** **0.04)**	Haematoma VKA vs. VKA and ASA 4 vs. 32% **(*****P*** **<** **0.05)**
CASPAR (Clopidogrel and Acetylsalicylacid in Bypass Surgery for Peripheral Arterial Disease) (23)	PAD after bypass surgery (below the knee) *N* = 851	Clopidogrel 75 mg o.d. vs. placebo in addition to ASA 75–100 mg	Graft occlusion, index bypass revision, index leg amputation Clopidogrel vs. placebo HR [95% CI] = 0.98 (0.78–1.23) (*P* = ns)	Moderate or severe bleeding Clopidogrel vs. placebo HR [95% CI] = 2.84 [1.32–6.08] **(*****P*** **=** **0.007)**
Vascular Outcomes Study of Aspirin along with Rivaroxaban in Endovascular or Surgical Limb Revascularization for PAD (VOYAGER PAD) (9, 24, 25)	After endovascular or surgical revascularization *N* = 6,564	ASA 100 mg o.d. or Rivaroxaban 2.5 mg b.d. and ASA 100 mg	MACE, acute limb ischemia or amputation ASA + rivaroxaban vs. ASA HR [95% CI] = 0.85 [0.76–0.96] **(*****P*** **=** **0.009)**	Major bleeding (ISTH) ASA + rivaroxaban vs. ASA HR [95% CI] = 1.42 [1.10–1.84]; **(*****P*** **=** **0.007)**

Significant P-values are displayed in bold.

PAD, peripheral arterial disease; CVD, cardiovascular disease; MI, myocardial infarction; ASA, acetylic salicylic acid; VKA, vitamin K antagonists; MACE, Major adverse cardiovascular event; MALE, Major adverse limb event. RR, relative risk; HR, hazard ratio; OR, odds ratio; CI, confidence interval; ICH, intracranial hemorrhage; GIB, gastrointestinal bleeding; o.d., omne in die, once daily; b.d., bis in die, twice daily.

**Table 2:** Guideline recommendations [according to (2, 26, 27).

**Clinical situation**	**Recommendation**	**Treatment duration**
Asymptomatic PAD without other clinical manifestations of atherosclerosis	No antiplatelet therapy	Since clinical situation changes (for example newly diagnosed cardiovascular disease)
Symptomatic PAD and low bleeding risk	Rivaroxaban 2.5 mg b.d. and ASA 100 mg o.d.	Long-term, regular assessment of bleeding risk
Symptomatic PAD and high bleeding risk	Clopidogrel 75 mg o.d. or alternatively ASA 100 mg o.d.	Long-term
After endovascular revascularization and low bleeding risk	Rivaroxaban 2.5 mg b.d. and ASA 100 mg o.d.	Long-term, if temporary addition of clopidogrel 75 mg duration shout be less than 30 days
After endovascular revascularization and high bleeding risk	Clopidogrel 75 mg o.d. or alternatively ASA 100 mg o.d.	Long-term, if temporary addition of clopidogrel as short as possible
After surgical revascularization and low bleeding risk*	Rivaroxaban 2.5 mg b.d. and ASA 100 mg o.d.	Long-term, regular assessment of bleeding risk
After surgical revascularization and high bleeding risk*	Clopidogrel 75 mg o.d. or alternatively ASA 100 mg o.d.	Long-term
PAD patients with indication for anticoagulation	Anticoagulation only	Long-term

^*^In some cases VKA can be discussed after venous grafts and ASA plus Clopidogrel after prosthetic grafts.

o.d.,omne in die, once daily; b.d., bis in die, twice daily.

## Stable symptomatic PAD

### Single antiplatelet therapy

Antiplatelet drugs reduce the occurrence of cardiovascular events in PAD and have a high level of recommendation in current guidelines (2, 3, 26, 27). The Antithrombotic Trialists' Collaboration Collaborative published a meta-analysis of randomized trials including antiplatelet therapy for prevention of death, myocardial infarction, and stroke in high-risk patients. Compared to placebo single antiplatelet therapy significantly improved prognosis in different atherosclerotic diseases including PAD (28).

In the CAPRIE (Clopidogrel vs. Aspirin in Patients at Risk of Ischaemic Events) study, clopidogrel was superior to ASA in the reduction of cardiovascular events (cardiovascular death, myocardial infarction, stroke) in patients with PAD (*N* = 6,452; mean age 65 years, 22% female, 28% diabetes) (11).

The Examing Use of Ticagrelor in Peripheral Artery Disease (EUCLID, mean age 66 years, 28% female, 38% diabetes) study compared ticagrelor with clopidogrel (12). In total 13,885 patients with symptomatic PAD (5% CLI) over the age of 50 were included. Patients with clopidogrel resistance were excluded. There was no advantage of ticagrelor compared to clopidogrel regarding cardiovascular events. Bleeding risk was comparable in both grups but patients in the ticagrelor group suffered more often from dyspnea.

### Combination of different antiplatelet drugs

The combination of ASA and clopidogrel was investigated in the CHARISMA (Clopidogrel for High Atherothrombotic Risk and Ischemic Stabilization, Management and Avoidance) study (13). While subgroup analysis of 3,096 PAD patients (2,838 symptomatic PAD, mean age 66 years, 30% female, 36% diabetes) showed a trend toward a slightly reduced rate of cardiovascular events the combination of ASA and clopidogrel was associated with an increase in minor bleeding complications. Major bleeding was not different between both groups.

Another study investigated the antiplatelet agent vorapaxar, which acts on platelets by inhibiting PAR-1 (Protease-Activated Receptor 1). In the Thrombin Receptor Antagonist in Secondary Prevention of Atherothrombotic Ischemic Events (TRA 2P)–Thrombolysis in Myocardial Infarction (TIMI) 50 trial, 26,449 patients were enrolled after myocardial infarction, with PAD or after stroke (29). The stroke arm was early terminated because of an excess of bleeding complications. A subgroup analysis of 3,787 patients with PAD (mean age 66 years, 29% female, 36% diabetes) showed no reduction in cardiovascular events but a reduction in limb events (14). However, because patients received vorapaxar in addition to monotherapy or dual platelet inhibition, there were significantly more bleeding events.

The PEGASUS (Prevention of Cardiovascular Events in Patients with Prior Heart Attack Using Ticagrelor Compared to Placebo on a Background of Aspirin) study showed beneficial effect of long-term use of ticagrelor and aspirin more than 12 months after acute myocardial infarction (30). In this trial there was also a significant reduction of cardiovascular events for the subgroup of patients with PAD (mean age 65 years, 22% female, 28% diabetes) (15).

### Combination of anticoagulation and antiplatelet drugs

The combination of antiplatelet and anticoagulant drugs for PAD patients was tested with the combination of ASA and warfarin in the WAVE (Warfarin Antiplatelet Vascular Evaluation Trial, mean age 64 years, 26% female, 27% diabetes) study (16). Warfarin was used in full dosage with a target INR between 2 and 3. The WAVE study failed because there was an increase in bleeding events without prognostic advantage.

Low dose rivaroxaban combined with ASA was first evaluated in the ATLAS ACS (Anti-Xa Therapy to Lower CV Events in Addition to Standard Therapy in Subjects with Acute Coronary Syndrome) TIMI 51 trial (31). In this study more than 15,000 patients after acute coronary syndrome were included and treated with rivaroxaban 2.5 mg or 5 mg twice daily compared to placebo in combination with ASA 100 mg. Rivaroxaban significantly reduced risk for MACE and stent thrombosis. There were more major bleeding events in the rivaroxaban groups, but no increase in fatal bleeding was found.

In the COMPASS (Cardiovascular OutcoMes for People Using Anticoagulation StrategyS) study, therapy with ASA 100 mg was compared to rivaroxaban 5 mg twice daily or the combination of rivaroxaban 2.5 mg twice daily and ASA 100 mg (17). The COMPASS study included 27,395 stable patients with CAD, PAD or carotid artery stenosis (CAS). Patients with indication for full dosage anticoagulation were excluded and in patients with new onset atrial fibrillation the study ended prematurely. The study was early stopped because of a significant superiority of combination therapy compared to ASA 100 monotherapy.

The combination of 2.5 mg rivaroxaban twice daily and ASA 100 mg significantly reduced both the combined primary endpoint (“MACE” = “Major Adverse Cardiovascular Events” = cardiovascular death, myocardial infarction, stroke) and all-cause mortality. There was an increase in severe (“major”) bleeding complications. These events were primarily gastrointestinal bleeding. Intracerebral or fatal bleeding complications did not occur more frequently in combination therapy.

The separate evaluation of patients with peripheral manifestation of atherosclerosis was predefined in COMPASS (8). This group (*N* = 7,470) includes patients with symptomatic PAD, with CAS or with CAD and low ABI (Ankle-Brachial Index). In addition to the primary endpoint of MACE events, an endpoint MALE was also defined. The MALE endpoint included ischemia-related major amputation or acute severe ischemia, which led to intervention (angioplasty, bypass surgery, thrombolysis, amputation). In patients with peripheral atherosclerosis, there was a significant reduction in both MALE and MACE in patients with combined therapy of rivaroxaban and ASA. As expected, the combination therapy results in a significant increase in major bleeding compared to placebo, but the positive effects outweigh these side effects.

In conclusion, the COMPASS study showed a significant reduction of cardiovascular and limb events with the combination of low dose rivaroxaban and ASA in patients with atherosclerosis in different vascular territories. Patients with PAD had higher thrombotic risk and greater advantage from more intensive antithrombotic medication (32). Therefore, recent guidelines recommend the combination of ASA and low rivaroxaban especially for patients with high risk for cardiovascular events and low bleeding risk (26, 27).

## Asymptomatic PAD

While there is a clear recommendation for the administration of antiplatelet drugs in symptomatic PAD, these drugs are not recommended in asymptomatic PAD patients. Two studies examined the administration of ASA compared to placebo in asymptomatic patients with decreased ankle brachial index (ABI) (33, 34). In the POPADAD (Prevention Of Progression of Arterial Disease And Diabetes) study patients with an ABI of 0.99 and less and in the AAA (Aspirin for Asymptomatic Atherosclerosis) trial patients with an ABI of 0.95 and less have been included. Both studies were unable to show an advantage of ASA compared to placebo, therefore current guidelines do not recommend antiplatelet therapy in patients with asymptomatic PAD without further manifestations of atherosclerosis (2, 26, 27). But due to the high coincidence between PAD and CAD or CAS, this constellation is certainly rare, and most patients will have other indications for antithrombotic therapy.

## After peripheral revascularization

Peripheral revascularization is indicated in patients with critical limb ischemia or intermittent claudication and short walking distance (2, 27). Endovascular revascularization is indicated when technically possible. The alternative is surgical revascularization using peripheral bypass operation. After peripheral revascularization, the risk for graft occlusion and acute limb ischemia is increased. Therefore, antithrombotic drugs are an important part of post-interventional therapy (6, 7).

### After endovascular revascularization

Numerous techniques, and devices such as drug-coated balloons or different stent designs are used for endovascular revascularization of PAD patients. However, patency of the treated vascular segment depends not only on the interventional method, but also on localization (aorto-iliacal, femoro-popliteal or below the knee arteries) and lesion morphology (long occlusion vs. short stenosis, degree of calcification). The choice, duration and dosage of antithrombotic therapy is therefore differently handled after peripheral intervention. Dual antiplatelet therapy with ASA 100 mg and clopidogrel 75 mg is widely used for 1 to 3 months. This common practice is recommended in current guidelines based on an expert consensus (2).

The randomized controlled CAMPER (Clopidogrel and Aspirin in the Management of Peripheral Endovascular Revascularization) study was planned to compare ASA monotherapy with dual antiplatelet inhibition. Unfortunately, the study had to be discontinued without result due to a lack of patient recruitment.

The MIRROR (Management of peripheral arterial interventions with mono or dual antiplatelet therapy) study compared a 6-month dual therapy with ASA and clopidogrel with ASA monotherapy (18). During the 6-month therapy, patients with dual platelet inhibition were less likely to have re-interventions at the treated vessel (TLR = target lesion revascularization). However, this advantage was no longer demonstrable after 12 months follow-up.

Cilostazol is approved as a drug to improve walking distance in intermittent claudication, but it also acts as a antiplatelet. The STOP-IC (The Sufficient Treatment of Peripheral Intervention by Cilostazol) study included a total of 200 patients with femoro-popliteal interventions (19). Patients received cilostazol or placebo in addition to ASA after endovascular revascularization. Patients with stents additionally received thienopyridine for 1 month regardless of randomization. Cilostazol was able to significantly reduce angiographic restenosis compared to placebo.

Another study investigated the administration of edoxaban with clopidogrel in addition to basic therapy with ASA for a period of 3 months in 203 patients after femoro-popliteal endovascular revascularisation (20). Edoxaban was used in full dosage. A comparable bleeding rate was found with a non-significant trend toward fewer restenosis under edoxaban therapy.

### After surgical revascularization

During the first year after surgical revascularization the risk for thrombotic bypass occlusion is increased. The risk depends on bypass material, vessel diameter, and location of distal anastomosis. In general, venous bypasses, especially when using the great saphenous vein, have better patency than prosthetic bypasses.

The Dutch BOA (the Dutch Bypass Oral anticoagulants or Aspirin) study compared ASA with VKA for peripheral venous or prosthetic bypasses in 2,960 patients (21). After prosthetic bypass patency was better with ASA after venous bypass VKA (target INR 3–4.5) was superior. A study by Sarac et al., was also able to show better patency of venous bypasses with VKA (target INR 2–3) compared to ASA (22). However, in both studies there was an increase in bleeding complications using VKA.

In the CASPAR (Clopidogrel and Acetylsalicylacid in Bypass Surgery for Peripheral Arterial Disease) study, ASA monotherapy was compared with dual antiplatelet therapy using ASA and clopidogrel in 851 patients after below the knee bypass grafting (23). The results of both therapy arms were comparable. Only in patients with prosthetic bypass there was a slight advantage for the dual antiplatelet therapy compared to ASA alone.

### Combination of ASA and low dose rivaroxaban after peripheral revascularization

The VOYAGER PAD (Vascular Outcomes Study of Aspirin along with Rivaroxaban in Endovascular or Surgical Limb Revascularization for PAD) is the largest randomized trial ever to evaluate anticoagulant therapy after peripheral revascularization (9). The VOYAGER PAD trial tested the combination of rivaroxaban 2.5 mg twice daily and ASA 100 mg compared to ASA alone. In total 6,564 patients were enrolled within 10 days after successful peripheral revascularization. One third of the patients had surgical and two third had endovascular revascularization. Most patients had intermittent claudication at time of inclusion, 24% of the patients were enrolled with critical limb ischemia.

After 3 years, the primary combined cardiovascular and peripheral efficacy endpoints (acute limb ischemia, amputation, myocardial infarction, stroke, cardiovascular death) were significantly reduced by combination therapy with rivaroxaban (ASA + rivaroxaban = 17.3% vs. ASA = 19.9%). The result was primarily influenced by a reduction in acute limb ischemia (ASA + rivaroxaban = 4.7% vs. ASA = 6.9%). The primary safety endpoint (TIMI major bleeding) was higher in the rivaroxaban group (2.7%) compared to the placebo group (1.9%, *P* = 0.07). However, the rate of fatal or intracerebral bleeding was not increased. There was no relevant difference regarding the primary endpoint comparing patients with intermittent claudication or critical limb ischemia [Hazard Ratio (95% Confidence Interval) 0.86 (0.74–0.99) or 0.85 (0.69–1.05)].

In comparison of thrombotic and bleeding risk, the advantage of a dual pathway inhibition predominates. Based on a population of 10,000 patients, the additional administration of rivaroxaban 2 × 2.5 mg prevents 181 primary endpoints per year at the cost of 29 severe bleeding complications.

Additional treatment with clopidogrel 75 mg was allowed for up to 6 months. There was no significant difference between treatment groups in the subgroup of patients using clopidogrel 75 mg (25). But patients who received clopidogrel on top of ASA and low dose rivaroxaban for more than 30 days had increase of major bleeding complications.

## Patients with indication for anticoagulation

Usually, anticoagulant drugs are necessary in patients with venous thromboembolism or atrial fibrillation. In these indications therapeutic dosage of anticoagulants is necessary. When VKA are used dosage is assessed according to INR ratio. Usually, a range of 2 to 3 in venous thromboembolism or atrial fibrillation is indicated. In these indications factor Xa inhibitors (rivaroxaban 20 mg o.d., apixaban 5 mg b.d., edoxaban 60 mg o.d.) or thrombin antagonists (dabigatran 110 or 150 mg b.d.) are used in standardized dosage if there is no renal insufficiency.

Recent data including patients with atrial fibrillation showed no reduction of thromboembolic events but an excess of bleeding complications when ASA is added to VKA (35). Therefore, current guidelines do not recommend additional antiplatelet therapy in patients with PAD, CAD or CAS and indication for full dosage anticoagulation (2, 26, 27). Only after coronary or peripheral intervention, temporarily addition of antiplatelet drugs is indicated (2, 26, 27). But the risk of bleeding must be considered, and the duration of combined therapy should be as short as possible.

## Assessment of bleeding risk

The individual bleeding risk should always be considered based on the current data situation and an individual benefit-risk assessment must be carried out. Compared to patients with CAD bleeding risk is increased in PAD patients but score like the HAS-BLED score are not assessed for PAD patients (26, 27, 36). A current consensus document concludes factors associated with bleeding risk in patients with PAD from different studies such as female gender, increased age, diabetes, arterial hypertension, smoking, hypercholesterinemia, bleeding history (for example peptic ulcer disease) and concomitant therapies (antithrombotic drugs, NSAID, beta-blockers) (26).

## Conclusion

Antithrombotic therapy is very important for patients with PAD, as it improves the prognosis of these high-risk patients. Antiplatelet therapy is indicated in all patients with symptomatic PAD. The COMPASS study showed a significant reduction of cardiovascular and limb events with the combination of low dose rivaroxaban and ASA. In the VOYAGER PAD study MACE and MALE events were significantly reduced by this combination therapy in patients after peripheral revascularization. Bleeding risk must be kept in mind especially for gastrointestinal bleeding in the first months of treatment, but for the longtime beneficial effects overweigh the initial risk. Therefore, recent guidelines recommend the combination of ASA and low rivaroxaban especially for patients with high risk for cardiovascular events and low bleeding risk (2, 26, 27). In patients with high bleeding risk single antiplatelet therapy with clopidogrel or alternatively ASA is recommended. In contrast, patients with asymptomatic PAD and no other manifestations of atherosclerosis do not need antiplatelet drugs. Finally patients with indication for full dosage anticoagulation because of concomitant atrial fibrillation or venous thromboembolism have no need for addition of an antiplatelet therapy. In addition to antithrombotic therapy, risk factor management such as consistent cessation of smoking and statin therapy are important to improve the prognosis of high-risk PAD patients.

Future direction includes the development of risk assessment models to stratify ischemic and bleeding risk in patients with PAD and to evaluate new antithrombotic regimes which may improve efficacy and safety for patients with PAD. Moreover, the role of interesting therapeutic approaches with focus on anti-inflammatory treatment have to be addressed for PAD patients.

## Author contributions

CE-K: original draft preparation. GW, VS, MS, and TM: writing–review and editing. All authors have read and agreed to the published version of the manuscript.

## Conflict of interest

Author CE-K received honoraria for lectures from Bayer, Boehringer Ingelheim, Bristol-Myers Squibb, Daiichi Sankyo, Pfizer, and Sanofi-Aventis. The remaining authors declare that the research was conducted in the absence of any commercial or financial relationships that could be construed as a potential conflict of interest.

## Publisher's note

All claims expressed in this article are solely those of the authors and do not necessarily represent those of their affiliated organizations, or those of the publisher, the editors and the reviewers. Any product that may be evaluated in this article, or claim that may be made by its manufacturer, is not guaranteed or endorsed by the publisher.

## Nomenclature

ASA, Acetylsalicylic acid; CAD, coronary artery disease; CAS, carotid artery stenosis; MACE, Major adverse cardiovascular event; MALE, Major adverse limb event; o. d., omne in die, once daily; b. d., bis in die, twice daily; PAD, Peripheral arterial disease; VKA, Vitamin K antagonist.
